# Liver Fibrosis in Non-Alcoholic Fatty Liver Disease and Progression to Hepatocellular Carcinoma in Patients with Inflammatory Bowel Disease: A Systematic Review

**DOI:** 10.3390/cancers15133367

**Published:** 2023-06-27

**Authors:** Samuel J. Martínez-Domínguez, Sandra García-Mateo, Viviana Laredo, Carla J. Gargallo-Puyuelo, Beatriz Gallego Llera, Julia López de la Cruz, Fernando Gomollón

**Affiliations:** 1 Department of Gastroenterology, Lozano Blesa University Hospital, 50009 Zaragoza, Spain; 2 Aragón Health Research Institute (IIS Aragón), 50009 Zaragoza, Spain; 3 School of Medicine, University of Zaragoza, 50009 Zaragoza, Spain; 4 CIBER for Liver and Digestive Diseases (CIBERehd), 28029 Madrid, Spain

**Keywords:** liver fibrosis, cirrhosis, NAFLD, hepatocellular carcinoma, inflammatory bowel disease, ulcerative colitis, Crohn’s disease

## Abstract

**Simple Summary:**

Non-Alcoholic Fatty Liver Disease (NAFLD) is a prevalent extraintestinal manifestation in patients with Inflammatory Bowel Disease (IBD). Liver fibrosis is the determinant long-term prognostic marker in patients with NAFLD because it is associated with progression to liver cirrhosis and hepatocellular carcinoma. In fact, NAFLD is already the most common liver disease worldwide and is becoming a leading cause of liver-related mortality. In this article, we systematically reviewed the available evidence regarding the prevalence and risk factors of liver fibrosis in patients with NAFLD and IBD, and we discussed the role and pathways of progression from liver fibrosis to hepatocellular carcinoma.

**Abstract:**

The aim of the systematic review is to assess the prevalence and risk factors of liver fibrosis in patients with Inflammatory Bowel Disease (IBD) and Non-Alcoholic Fatty Liver Disease (NAFLD) and to discuss the role of liver fibrosis in the progression to hepatocellular carcinoma (HCC). We performed a structured search in PubMed, Web of Science, Embase, and Scopus up to 3 March 2023 to identify observational studies reporting liver fibrosis in patients with NAFLD and IBD. Quality of studies was assessed using the Newcastle-Ottawa Scale (NOS) score. A total of 23 studies met our inclusion criteria, including 629,781 patients. A total of 10 cross-sectional, 3 case-control, and 10 cohort studies were included. Fourteen studies had a NOS score ≥ 7 points. NAFLD was diagnosed in 2162/6332 (34.1%) IBD participants. However, NAFLD diagnosis was established in 924/2962 (31.2%) healthy individuals without IBD. Advanced liver fibrosis was found in 116 (11.6%) of 992 IBD patients with NAFLD. Most studies found an association between NAFLD and classic cardiovascular risk factors such as older age, male sex, higher BMI, diabetes, hypertension and dyslipidemia. In addition, metabolic syndrome features were also associated with an increased risk of significant and advanced liver fibrosis. Although no strong association between NAFLD and IBD therapy was reported, some studies associated NAFLD with IBD diagnosis, Crohn’s Disease, a complicated course of IBD, disease activity, and IBD duration. Advanced liver fibrosis was also associated with Crohn’s disease in several studies. In conclusion, NAFLD and advanced liver fibrosis are prevalent and clinically relevant extraintestinal manifestations, so its diagnosis and potential progression to HCC should be carefully considered in daily clinical practice.

## 1. Introduction

Non-Alcoholic Fatty Liver Disease (NAFLD) is defined as the presence of more than 5% of steatosic hepatocytes in patients without significant alcohol intake [1]. Recently, some authors adopted a new term named Metabolic Associated Fatty Liver Disease (MAFLD), based on the concomitant presence of liver steatosis and metabolic risk factors [2]. The disease encompasses a broad histological spectrum, from steatosis without evidence of inflammation (Non-Alcoholic Fatty Liver: NAFL) to steatosis with necro-inflammatory injury (Non-Alcoholic Steatohepatitis: NASH), which can progress to liver cirrhosis and hepatocellular carcinoma (HCC) [3]. In patients with inflammatory bowel disease (IBD), a chronic condition characterized by an exaggerated immune response mainly involving the gastrointestinal tract, NAFLD is a common extra-intestinal manifestation [4]. In fact, a retrospective study including patients with IBD reported that NAFLD was the most common cause of abnormal liver test after histological evaluation (32.7%) [5].

The worldwide burden of NAFLD is increasing with a global prevalence of approximately 25% and it is becoming a leading cause of chronic liver disease and liver transplantation in the United States [3,6]. The prevalence can rise to 55% in patients with type 2 diabetes mellitus and to 75% in obese population [7,8]; however, it should be noted that up to 40.8% of patients with NAFLD are not obese [9]. The prevalence of NAFLD in IBD population range from 2.4% to 52%, probably due to methodological differences [10,11,12,13]. In a systematic review conducted in 2014, the overall prevalence of fatty liver in IBD was 23% [4], while a meta-analysis including 19 studies found a prevalence of 27.5% with higher prevalence in studies published after 2015 [14], and in another one the prevalence rose to 30.7% [15]. In a recently published case-control study including 831 IBD patients, the prevalence of MAFLD was significantly higher in IBD than in general population (42% vs. 32.7%; *p* < 0.001) and the presence of IBD was a risk factor for developing MAFLD [16].

Obese patients have an increased risk of developing NAFLD in the general population but also in IBD [1,13,14]. Nevertheless, studies in non-obese populations suggest that Body Mass Index (BMI) is not the only risk factor for NAFLD, so other metabolic conditions should also be taken into account [9]. In a meta-analysis including IBD patients, type 2 diabetes, hypertension, obesity, and insulin resistance were associated with an increased risk of NAFLD [14]. However, another study comparing IBD patients with NAFLD and non-IBD patients with NAFLD reported that IBD patients had less metabolic risk conditions, suggesting that IBD-related factors could also influence the risk of developing NAFLD [17]. In fact, NAFLD was an independent risk factor for atherosclerotic cardiovascular disease, regardless of classic metabolic risk factors [18,19,20].

Beyond cardiovascular risk, NAFLD is a clinically relevant condition due to the potential progression to NASH, advanced fibrosis, cirrhosis, and HCC [1]. About 10–20% of patients with NAFLD will develop cirrhosis, and the rate of disease progression in NASH is 0.09 per year with an annual incidence of HCC of 0.21 per 1000 person-year, being higher in patients with cirrhosis (10.6 per 1000 person-year) [21]. Comparing with non-inflammatory NAFLD, patients with NASH have a tenfold increased risk of developing HCC [22]. Although advanced fibrosis increases the risk of HCC, approximately 20% of patients with NAFLD HCC are not cirrhotic, suggesting the implication of different carcinogenesis pathways in this population [3,21]. In fact, the risk of HCC among non-cirrhotic liver disease is significantly higher in NASH compared to other causes [23]. In the IBD population, the prevalence of NAFLD with advanced fibrosis ranges from 8.1 to 13.6%, but the incidence and prevalence of HCC is not well established [11,12,15]. In a case-control study, the prevalence of advanced fibrosis in MAFLD was higher in those also affected by IBD (9.5% vs. 2.31%; *p* < 0.001) and IBD was an independent risk factor for advanced fibrosis [16]. The potential development of HCC at early fibrosis stages makes the diagnosis difficult and, currently, there are no screening programs in this population, so the neoplasm is usually diagnosed at advanced stages [24].

Due to the higher risk of NAFLD in IBD and the complex interaction between the two diseases, the aim of this systematic review is to assess the prevalence and risk factors of liver fibrosis in patients with IBD and NAFLD and to discuss the role of liver fibrosis in the progression to HCC.

## 2. Materials and Methods

This systematic review was conducted according to the Preferred Reporting Items for Systematic Reviews and Meta-Analyses (PRISMA) 2020 guidelines [25]. Institutional review board approval was not required due to the study design.

### 2.1. Data Sources and Search Strategy

We performed a comprehensive literature search in PubMed, Web of Science, Scopus and Embase up to 3 March 2023. Only human studies were included but no language or time restrictions were applied. Database searches were performed independently by three authors (S.J.M.D., S.G.M. and C.J.G.P) using the following terms: inflammatory bowel disease OR IBD OR ulcerative colitis OR UC OR Crohn’s disease OR CD. The Boolean operator “AND” was used to combine these terms with: NAFLD OR non alcoholic fatty liver disease OR NAFL OR non alcoholic fatty liver OR steatosis OR NASH OR steatohepatitis OR hepatosteatosis OR MAFLD OR metabolic associated fatty liver disease OR liver fibrosis OR liver cirrhosis. All references were imported into an EndNote 20.5 reference manager software file. Duplicate references were electronically removed and subsequently reviewed manually. Titles and abstracts were independently assessed by three authors (S.J.M.D., S.G.M. and V.L.). Later, full-text articles were reviewed for inclusion by three authors (S.J.M.D., S.G.M. and B.G.L.), and any disparities were discussed and resolved by consensus (F.G). Finally, references from eligible studies were also reviewed to identify potential studies missed by the electronic search.

### 2.2. Selection Criteria

The following inclusion criteria were applied: (1) studies including patients with established diagnosis of IBD; (2) studies assessing prevalence and/or risk factors of liver fibrosis in patients with NAFLD or MAFLD; and (3) observational studies, with or without control group.

Studies were excluded in case of: (1) absence of original data (reviews, systematic reviews, meta-analyses, letters, editorials, guidelines); (2) conference communications; (3) non-human studies; (4) studies involving only non-IBD patients or when IBD population data could not be identified; (5) absence of data regarding liver fibrosis in participants with NAFLD or MAFLD; and (6) studies focusing on patients with IBD receiving hepatotoxic drugs.

### 2.3. Data Extraction and Analyses

Each included study was critically reviewed by three authors (S.J.M.D., S.G.M., and V.L.) and disparities were resolved by consensus among all authors. The following data was extracted from the selected studies: first author, year of publication, study location, study design, sample size, age, gender, Body Mass Index (BMI), IBD type, IBD therapy, prevalence of NAFLD and liver fibrosis, and risk factors for NAFLD and liver fibrosis.

NAFLD was defined as the presence of liver steatosis after exclusion of significant alcohol consumption and secondary causes of fat accumulation in the liver [26]. MALFD was used based on the presence of liver steatosis plus one of the three following conditions: overweight/obesity, type 2 diabetes mellitus, or at least two metabolic risk abnormalities in case of lean/normal weight patients. Metabolic risk abnormalities were: waist circumference ≥ 102 cm in men and 88 cm in women, blood pressure ≥ 130/85 mmHg or specific drug treatment, plasma triglycerides ≥ 150 mg/dL or specific drug treatment, plasma HDL-cholesterol < 40 mg/dL for men and <50 mg/dL for women or specific drug treatment, prediabetes, homeostasis model assessment of insulin resistance score ≥ 2.5 and C-reactive protein > 2 mg/L [27,28].

The prevalence of NAFLD and liver fibrosis was calculated, including all studies with available information. Absolute frequencies were estimated when only relative frequencies were reported. Data from longitudinal studies were obtained from the beginning or end of follow-up depending on the study design and the available information (detailed information of each article in Supplementary Table S1), as any of the studies applied interventions to modify the natural history of liver disease. Abomhya 2022 was excluded in the numerical calculation of NAFLD prevalence as it reported a much lower prevalence than the rest of the studies and, given its large sample size compared to the other studies, this distorted the overall result.

### 2.4. Risk of Bias Assessment

Quality of observational eligible studies was assessed using the Newcastle-Ottawa Scale (NOS) by three authors (S.J.M.D., S.G.M. and J.L.C.). Disagreements were solved thorough discussion among all authors. Studies were considered to have low risk of bias if they achieved at least 7 stars. NOS for cohort and case-control studies is compound by 8 items: 4 items for the selection of exposed/non-exposed group or cases/controls groups, 1 item for the comparability of the groups, and 3 items for outcome evaluation/ascertainment of exposure. A maximum of 1 star can be assigned to each item except for comparability, which can be assigned 2 stars (maximum total score 9 stars) [29]. NOS scale adapted for cross-sectional studies have 7 items, categorized into three groups: 4 items for the selection of the groups, 1 item for comparability of the different outcome groups, and 2 items for the assessment of the outcome. A maximum of 1 star can be assigned to each item except for ascertainment of the exposure (selection), comparability, and assessment of the outcome (outcome section), with the maximum total score 10 stars [30].

## 3. Results

### 3.1. Study Characteristics

**Figure 1:**
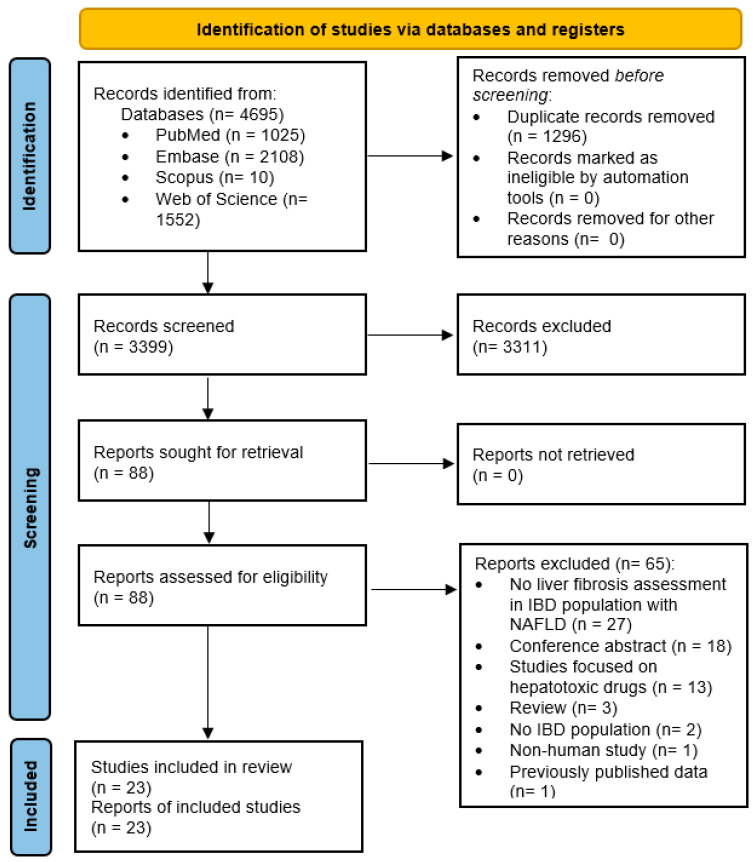
Flow diagram of the search process. IBD: Inflammatory Bowel Disease. NAFLD: Non-Alcoholic Fatty Liver Disease.

The flow diagram of study retrieval for the systematic review is detailed in Figure 1. A total of 4695 records were identified from databases (PubMed 1025, Embase 2108, Scopus 10, and Web of Science 1552). After electronically removing duplicate records, 3399 records were reviewed by title and abstract excluding 3311 of them. Eighty-eight full-texts were reviewed, excluding 65 additional reports being the most frequent reasons: lack of liver fibrosis assessment in IBD population with NAFLD (n = 27), conference abstracts (n = 18), and studies focused on patients receiving hepatotoxic drugs (n = 13). Finally, 23 studies were included in the systematic review [10,11,12,16,31,32,33,34,35,36,37,38,39,40,41,42,43,44,45,46,47,48,49].

All the included studies were observational, including 10 (43.5%) cross-sectional studies [11,12,32,34,36,38,39,40,46,48], 10 (43.5%) cohort studies [10,31,33,35,37,42,43,45,47,49], and 3 (13%) case-control studies [16,41,44] (Table S1). All studies were published between 2015 and 2023. Eleven (47.8%) were conducted in North America [10,11,12,32,33,34,35,38,39,41,42], ten (43.5%) in Europe [16,36,37,40,43,44,45,46,47,48] and two (8.7%) in Asia [31,49]. Only six (26.1%) studies had a control group composed by patients without IBD [16,32,36,37,41,44]. Five (21.7%) studies included only IBD patients with CD [10,11,12,41,43], but no studies enrolled only patients suffering from UC.

Regarding the diagnostic method for NAFLD, 3 performed a retrospective review of database records [10,35,42], 2 combined liver US and TE [48,49], 5 used US [31,36,39,40,44], 6 used TE [12,16,38,45,46,47], 2 used laboratory tests alone [33,37], 3 liver histology [32,34,43], 1 computed tomography [11], and 1 Magnetic Resonance [41]. Regarding the diagnostic method for liver fibrosis, two performed a retrospective review of database records [10,35], 10 used TE [12,16,36,38,40,45,46,47,48,49], seven used laboratory tests alone [11,31,33,37,39,41,42], two used liver histology [32,34], one used a combination of TE and laboratory tests [44], and one used a combination of TE and histology [43]. Therefore, NAFLD and liver fibrosis were assessed by non-invasive methods in 20 (86.9%) and 21 (91.3%) studies, respectively. Studies using TE for liver fibrosis assessment defined significant liver fibrosis as liver stiffness above 7 kPa in five (41.7%) studies [12,40,43,45,48], 7.2 kPa [16,46] in two (16.7%) studies, 7.3 kPa [47] in one (8.3%) study, 7.7 kPa in one (8.3%) study [36], ≥8 kPa [38,49] in two (16.7%) studies, and no specified in one (8.3%) study [44].

### 3.2. Participants’ Characteristics

A total of 629,781 participants were included, 220,128 (35%) with IBD and 409,653 (65%) without IBD at enrolment. CD was the most frequent IBD type with 218,162 (99.1%) participants, followed by UC with 1907 (0.9%) patients. Sample size ranged from 35 (0.01%) patients [43] to 405,413 (64.4%) patients [37]. The median/mean age ranged from 33 to 57 years, and 278,484 (44.2%) were males and 351,297 (55.8%) females. Population included by most studies had a mean BMI ≥ 25 kg/m^2^, except participants included by Bessisow 2016, Veltkamp 2022, and Yen 2021 [33,48,49]. Detailed BMI levels reported by Chen (2023), Magri (2019), and Palumbo (2018) for non-NAFLD population were lower [12,37,40]. Thiopurines use was reported in 12 studies with 544 participants (0.2% of IBD population) [12,16,33,36,39,40,42,43,44,45,46,48], and Methotrexate use in 7 studies including 54 participants (0.02% of IBD population) [12,16,33,36,39,45,48]. Six studies did not report detailed information about ongoing IBD therapy [10,11,32,35,37,41].

### 3.3. Risk of Bias Assessment

NOS scores for risk of bias assessment are shown in Tables S2–S4. Overall, fourteen (60.9%) studies had a NOS score ≥ 7 points [10,11,12,16,32,34,36,37,38,39,40,45,46,48] and the lowest score was 4 points [49]. Regarding cross-sectional studies, all of them had a NOS score ≥ 8 points. Only four (40%) studies achieved 2 stars for comparability [32,34,36,46] because most of them lacked a control group including comparable healthy controls. The patients included by Arggarwal (2022) and Bosch (2017) [32,34] came from a selected group who underwent liver biopsy, so the sample was not considered representative of IBD population. Only three (30%) cohort studies showed a NOS score ≥ 7 points [10,37,45] and, again, comparability and selection of the non-exposed cohort were the lowest-scoring items. Finally, Rodriguez-Duque (2022) was the only case-control study that achieved NOS ≥ 7 points [16].

### 3.4. Prevalence of NAFLD and Liver Fibrosis

NAFLD was diagnosed in 2162 (34.1%) patients of a total of 6332 IBD participants included in 18 studies [11,12,16,31,33,34,36,38,39,40,41,42,43,45,46,47,48,49]. NAFLD diagnosis was established in 924 (31.2%) of 2962 non-IBD participants from the control groups of two studies [16,41]. The prevalence of NASH was 36 (5.9%) of 608 IBD patients included in three studies [34,38,39].

Data on liver fibrosis were reported heterogeneously in the different studies. Advanced liver fibrosis was found in 116 (11.6%) of 992 IBD patients with NAFLD included in six studies [11,12,16,32,33,42]. Three studies [16,32,41] demonstrated a significantly higher prevalence of liver fibrosis in IBD patients with NAFLD compared with NAFLD individualswithout IBD. Aggarwal 2022 reported that 30% of patients with NAFLD and IBD and 17% of patients with NAFLD without IBD had advanced liver fibrosis [32]. On the other hand, Bessisow 2016 showed a 2.2% of advanced liver fibrosis in patients who developed NAFLD after a median follow-up of 3.2 years [33]. Chen 2023 conducted also a cohort study demonstrating an increased incidence of IBD in patients with NAFLD at high risk of advanced disease compared with non-NAFLD patients [37]. Cirrhosis ranged from 0% to 8–10.2% [10,34,39], and a greater proportion of cirrhosis was found in patients with IBD and concomitant primary sclerosing cholangitis. Finally, some studies did not specified between significant and advanced liver fibrosis [31,39] and other studies reported the mean values of liver stiffness or laboratory tests but not the prevalence [35,36,44,45].

### 3.5. Risk Factors for NAFLD and Liver Fibrosis in IBD Population

Most studies found an association between NAFLD and classic cardiovascular risk factors such as older age, male sex, higher BMI, type 2 diabetes, arterial hypertension, and dyslipidemia. In fact, Spagnuolo (2019) demonstrated that weight gain is a predictor of steatosis worsening [45]. Regarding IBD related risk factors, some studies associated NAFLD with the diagnosis of IBD [16], complicated course of IBD [16,46], disease activity [33,39], longer IBD duration [33,46,48], shorter IBD duration [49], or CD [34,37,41]. No strong association between NAFLD and IBD therapy was reported. In addition, Abomhya 2022 reported an increase in hospitalization costs and length during follow-up in discharged patients with CD and NAFLD [10]. Recently, Kablawi (2023) noticed that NAFLD, longer IBD duration, and UC were associated with intermediate-high cardiovascular risk [38].

In parallel, metabolic syndrome features were also associated with an increased risk of significant liver fibrosis [12,36,40,46] and advanced liver fibrosis [16]. In fact, Spagnuolo 2019 reported a significant lower liver stiffness in patients who lost weight after a mean follow-up of 4 years [45]. Regarding IBD related risk factors, several studies associated CD with significant liver fibrosis [12,41] and advanced liver fibrosis [32]. Particularly, Ribaldone (2015) found an association between intermittent CD course and significant liver fibrosis [43], whereas Carr (2017) found no influence of IBD extent or severity [35], Rodriguez-Duque (2022) found association between complicated course of IBD and advanced liver fibrosis [16], Trifan (2022) and Veltkamp (2022) reported an association between IBD duration and significant liver fibrosis [46,48], and Van Lingen (2021) observed an increased prevalence of significant liver fibrosis after 6–12 months of follow-up in patients with IBD activity [47]. Although Ritaccio (2021) found no effect of medication exposure [42], methotrexate [12] and thiopurines [43] use was associated with increased liver fibrosis in other studies. Kablawi (2023) found an association between significant LF and intermediate-high cardiovascular risk [38].

## 4. Discussion

This systematic review reported the prevalence and risk factors associated with NAFLD and liver fibrosis in patients with IBD. In the last few months, several articles focused on NAFLD and IBD [10,16,32,37,38,46,48], a topic of increasing interest due to its prognostic implications. We found that NAFLD is highly prevalent in IBD population (34.1%), consistent with data published by other systematic reviews (30–32%) [15,50]. Although we observed that NAFLD was also very prevalent in the general population (31.2%), it was less frequent compared to IBD population. Previous reviews reported a prevalence of NAFLD about 25% in general population, being significantly lower than the prevalence in IBD population. This finding was recently reported by Rodriguez-Duque (2022) in one of the largest cohorts of IBD population assessing MAFLD and liver fibrosis in patients with IBD [16]. We found higher prevalence of NAFLD in the general population compared to previous studies, and it could be explained because most studies lacked a control group composed of healthy non-IBD individuals, so only two had available data to estimate the prevalence in non-IBD population. Most studies identified that participants with classical metabolic syndrome features had increased risk of NAFLD development. The higher prevalence of NAFLD in patients with IBD compared to general population suggests that IBD itself could play a role in NAFLD development. This point was confirmed by multiple studies revealing an association between IBD, CD or IBD-related factors and the risk of NAFLD [16,33,34,37,39,41,46,48].

Due to the heterogeneous report of liver fibrosis status, we estimated a prevalence of advanced liver fibrosis of 11.6% including information from six studies [11,12,16,32,33,42]. This is consistent with data reported by a previous meta-analysis (13.6%) [15]. Apart from the classic cardiovascular risk factors, CD was associated with significant and advanced liver fibrosis. This finding supports the importance of considering the presence of both NAFLD and liver fibrosis in patients with IBD, especially in patients with CD. On the one hand, the increased cardiovascular risk can seriously affect the prognosis of the patient with IBD, who is increasingly older and has more comorbidity. On the other hand, the potential progression to cirrhosis or CHC may be also decisive for the long-term evolution of the patient.

Unfortunately, we did not find studies reporting the prevalence and/or risk factors of HCC specifically performed in the IBD population. However, a recent study assessed the effect of immune checkpoint in patients with pre-existing IBD, finding that approximately 40% patients experienced relapse of IBD with a rate of discontinuation of 35% [51]. Conversely, the beneficial chemopreventive effect of statins against HCC has been described, a widely used drug by patients with NAFLD given the frequent coexistence of metabolic syndrome [52].

Many studies have investigated the elements involved in fibrosis progression [1]. In this way, the presence of NASH increases the risk of fibrosis progression and liver mortality [53], as well as type 2 diabetes, that has been identified as one of the most important predictors of fibrosis and a risk factor for HCC [9,54]. In IBD population, older age and obesity are independent risk factors for liver fibrosis (aOR 1.38 and 1.14, respectively) [12]. In a study including 1009 patients with a diagnosis of NAFLD based on liver biopsy, Crohn’s disease but not ulcerative colitis was an independent risk factor for advanced liver fibrosis (aOR 4.09 and 0.29, respectively).

Genetic factors can also influence the risk of disease progression, fibrosis and HCC. The polymorphism p.I148M of the PNPLA3 gene has been linked to an increased risk of NAFLD, NASH, HCC, and liver-related mortality [55]. It is one of the most important factors related with negative outcomes in NAFLD patients [24]. In some studies, this variant has been associated with fibrosis progression and NASH, especially in homozygous [56,57,58,59]. It has also been related to an increased risk of HCC and mortality, with the highest risk in patients with both alleles affected [60,61,62]. In IBD patients, this genetic variant has been associated with an increased risk of fatty liver (OR 2.9) [63]. There is also a link between TM6SF2 and MBOAT7 variants and the risk of advanced fibrosis and HCC [55].

The development of HCC in non-cirrhotic patients with NASH suggest that other pathogenic ways independent of fibrosis can lead to carcinogenesis, so some studies have investigated the role of the microbiota, metabolic changes, and immune and fibrotic cells [64]. There is growing evidence suggesting that dysbiosis and gut permeability, which is increased in patients with IBD, allows pathogens and their products to be in contact with liver immune receptors (Kupffer cells and stellate cells) leading to a pro-inflammatory state with liver injury and restorative mechanisms [65]. There are some similarities in gut microbiota changes related to both, NAFLD and IBD [66]. Some studies suggest that Gram negative bacteria, which are more common in NAFLD and obese patients, can increase the risk of liver fibrosis, and this may be associated with some wall components (lipopolysaccharides) [65,67]. Loomba et al. found a specific microbiome composition related to advance fibrosis in NAFLD [67]. In this population, Firmicutes and Bacteroidetes are increased, however, in cases of advanced fibrosis gram negatives as Proteobacteria phylum increases, while Gram-positive (for example, Firmicutes) decrease. In another study, Ruminococcus was associated with advanced fibrosis, probably due to their capacity to produce alcohol, which can damage gut integrity and led to liver inflammation [68].

Bile acids are produced in the liver through cholesterol oxidation, secreted into the duodenum and conjugated by gut microbiota in order to facilitate the absorption of fatty acids and also to activate the Farnesoid X Receptor (FXR) of the enterocytes, which regulates the enterohepatic circulation and play an important role in the glucose metabolism [66]. Dysbiosis leads to changes in bile acids composition and decreases the activation of FXR with an increase in serum bile acids and intestinal permeability, changes in lipid and glucose metabolism, and, also, a pro-inflammatory response [65]. In IBD, it is also associated with a disbalance in the Th17 response [66]. Moreover, bacterial dysbiosis has also been associated with higher levels of methylamines (TMAO) and hepatic accumulation of triglycerides with a higher risk of NALFD and carcinogenesis [65,69].

Concerning the role of metabolic changes, the development of NASH is secondary to the accumulation of free fatty acids in the liver with an increase in the gluconeogenesis, which leads to higher levels of glucose and more conversion to fatty acids [70,71]. When fatty acids exceed liver capacity, lipotoxicity leads to mitochondrial damage, oxidative stress, hepatocyte injure, and pro-inflammatory response, as well as the activation of hepatic stellate cells, increasing the production of fibrotic compounds [70]. Tissue damage activates platelets that are also responsible for pro-inflammatory and fibrotic response, and in animals, inhibition of platelet activation has been associated with a decreased risk of HCC [72]. The oxidative stress and inflammatory response contribute to the genomic instability and mutagenesis and the risk of HCC. Insulin resistance and the production of pro-inflammatory cytokines can increase the hepatocyte proliferation, with mutagenesis accumulation. Activated stellate cells are important in the tumor environment and can play a role avoiding the immune surveillance in HCC, so it could be useful as a therapeutic target [73].

Innate but also adaptive immunity seem to play an important role in the development of fibrosis and HCC, since in NAFLD the continuous inflammatory response ends up producing dysregulation of the immune system with immunosuppression and tumor tolerance. Monocyte-derived macrophages form complexes with dead steatotic hepatocytes triggering a pro-fibrotic response, which could be involved in carcinogenesis [22]. Neutrophils with a N2 phenotype are more common in HCC and have a negative effect against T-cells leading the tumor to escape from immune surveillance [22]. Natural Killers (NK) are also associated with progression of NAFLD and some studies have demonstrated an altered phenotype of NK in HCC, reducing the cytotoxicity and oncologic surveillance [74]; in fact, fibroblastic cells in HCC can inhibit the activity of NK [75].

In conclusion, liver fibrosis is an independent risk factor for HCC but NAFLD HCC can also occur in absence of cirrhosis, so other elements should be considered in this population [24,76,77]. Metabolic conditions, such as diabetes, obesity, and metabolic syndrome, are also independent risk factors for HCC [24,64]. In many studies, the presence of type 2 diabetes is the main factor associated with an increased risk of HCC, being higher in patients with a longer evolution of diabetes [77,78,79]. An appropriate glycemic control seems to decrease the risk of HCC; however, the effect of each diabetes therapy is contradictory, since some studies have found a benefit for metformin but not for insulin or sulfonylureas [64,80,81,82]. In this way, the presence of metabolic syndrome and diabetes increases by five times the risk of HCC [24]. Obesity has also been associated with an increased risk of HCC and recurrence in cirrhosis, especially in patients with other metabolic alterations [24]. These findings are particularly important as these conditions often coexist in IBD patients with NAFLD.

Finally, we acknowledge the following limitations of our study. First, the different study designs of included studies and the high rate of uncontrolled studies. Second, the heterogeneity of diagnostic methods and cut-offs used for NAFLD and liver fibrosis diagnosis. Third, the variability of data reported by each study, especially for liver fibrosis. Fourth, the absence of solid evidence on the prevalence and specific risk factors of HCC in patients with concomitant IBD and NAFLD. Nevertheless, the strengths of the study are its large sample size, the high rate of recently published articles and that, on average, the quality of the included studies was high. Our findings are clinically relevant as they reveal the magnitude of a comorbidity, whose adequate prevention and treatment could improve the prognosis and quality of life of the IBD population.

## 5. Conclusions

NAFLD is a prevalent extraintestinal manifestation in patients with IBD and it can promote HCC thorough liver fibrosis development, although not exclusively. The presence of advanced liver fibrosis is not rare in the IBD population with NAFLD, especially in those suffering from Crohn´s Disease. Therefore, the presence of NALFD should be considered in patients with IBD, especially in association with additional risk factors. After diagnosis, hygienic-dietary measures should be initiated and evolution should be monitored to prevent complications, such as cirrhosis or HCC. Future studies are needed to evaluate the prevalence and specific risk factors of HCC in IBD population with NAFLD.

## Data Availability

Not applicable.

## Supplementary Materials

The following supporting information can be downloaded at: https://www.mdpi.com/article/10.3390/cancers15133367/s1, Table S1: Main characteristics of the 23 observational studies included in the systematic review. Table S2: Risk of bias assessment of the 10 cross-sectional studies included in the systematic review according to the Newcastle-Ottawa Scale (NOS). Table S3: Risk of bias assessment of the 10 cohort studies included in the systematic review according to the Newcastle-Ottawa Scale (NOS). Table S4: Risk of bias assessment of the 3 case-control studies included in the systematic review according to the Newcastle-Ottawa Scale (NOS).
