# Supplementary material for: Liver Fibrosis in Non-Alcoholic Fatty Liver Disease and Progression to Hepatocellular Carcinoma in Patients with Inflammatory Bowel Disease: A Systematic Review
Source: Cancers (Basel). 2023 Jun 27;15(13):3367. doi: 10.3390/cancers15133367 (PMC10340150; doi:10.3390/cancers15133367)
Supplement: Supplementary file 1 [file cancers-15-03367-s001.zip › Table S1 Characteristics of included studies 5 april 2023.pdf]

Table S1. Main characteristics of the 23 observational studies included in the systematic review

**First author, year**	**Study location**	**Study design**	**Samplesize**	**Diagnostic method**	**Age** (years)	**Gender** (male) n (%)	**BMI** (Kg/m^2)	**IBD type** n (%)	**Ongoing IBD therapy** n (%)	**NAFLD prevalence** n (%)	**Risk factors for NAFLD**	**Liver fibrosis prevalence** n (%)	**Risk factors for liver fibrosis**
**Abomhya, 2022 [10]**	Multicentric, USA	Nationwide retrospective cohort study<br><br>Follow-up: All-cause 30 day non-elective readmission	All patients CD: 215,049.<br><br>At baseline:<br>●CD + NAFLD: 5,268<br>●CD but No-NAFLD: 209,781	Record review of national database of discharged patients with CD	●NAFLD: 53 (42-63)<br>●No-NAFLD: 53 (36-68)	●NAFLD: 2,139 (40.6)<br>●No-NAFLD: 88,737 (42.3)	Obesity (≥30 Kg/m^2):<br>●NAFLD: 1,544 (29.3)<br>●No-NAFLD: 23,705 (11.3)	CD 215,049 (100)<br>UC 0 (0)	NA	At baseline:<br><br>NAFLD: 2.4%<br>NASH: 0.42%	●Age 45-64 years<br>●Female<br>●Metabolic syndrome<br>●Obesity<br>●Diabetes<br>●Hypertension<br>●Dyslipidemia<br>NAFLD increase hospitalization costs and length. NASH increase mortality and readmission	At baseline:<br><br>Cirrhosis: 537 (10.2)	NA
**Al-Mohannadi, 2020 [31]**	Unicentric, Doha, Qatar	Retrospective cohort study<br><br>Aim: developement of NAFLD during follow-up	IBD: 913	NAFLD: US<br>LF: NAFLD fibrosis score	36.9±13.2	550 (60.2)	26.9±6.1	CD 383 (41.9)<br>UC 530 (58.1)	Biologic agents: 279 (30.6)<br>Previous surgery: 166 (18.2)	During follow-up:<br><br>NAFLD: 108 (11.8)<br>Severe steatosis: 24 (22.2)	●Older age<br>●Higher BMI<br>●Diabetes<br>●Hypertension<br>●Anti-TNF no increase NAFLD risk	During follow-up:<br><br>LF: 19 (2.2)	ROC value of 0.7 for NALFD fibrosis score cut-off of ≥-1.67
**Aggarwal, 2022 [32]**	Unicentric, Cleveland, USA	Cross-sectional study<br><br>Medical records review of	●IBD + NAFLD: 50<br>●NAFLD without IBD: 959	Histology<br>●NAFLD: NASH-CRN criteria<br>●NASH: NAFLD activity	●IBD + NAFLD: 50.4 [39.7, 56.8]<br>●NAFLD without	●IBD + NAFLD: 19 (38)<br>●NAFLD without IBD: 364 (38)	●IBD + NAFLD: 32.5 [27.6, 39.7]<br>●NAFLD	CD 34 (68)<br>UC 16 (32)	NA	NASH<br>●IBD + NAFLD: 31 (62)<br>●NAFLD without IBD: 537	-	Any grade of LF:<br>●IBD + NAFLD: 35 (70)<br>●NAFLD without IBD: 604 (63)	Advanced LF:<br>●CD (CD with advanced LF were older and with higher prevalence of diabetes)

Table S1. Main characteristics of the 23 observational studies included in the systematic review

		1,009 patients with histologically proven NAFLD		score 3 + any stage LF<br>●LF: Kleiner staging system (advanced LF: III-IV)	IBD: 50.7 [39.9,58.7]		without IBD: 40.2 [33.3, 47.4]			(56)<br>p=0.4		p=0.003<br>Advanced LF:<br>●IBD + NAFLD: 15 (30)<br>●NAFLD without IBD: 163 (17)<br>p=0.02	
**Bessisow, 2016 [33]**	Unicentric, Quebec, Canada	Retrospective cohort study<br><br>Median follow-up 3.2 years. Aim: developement of NAFLD during follow-up	IBD without NAFLD: 321	●NAFLD if HSI ≥ 36<br>●Advanced LF if FIB-4 ≥ 2.67	33.7 [25.1-46.9]	Male 151 (47)	22.9 [20.9, 24.7]	CD 217 (67.6)<br><br>UC 104 (32.4)	5-ASA 127 (39.6)<br>SE 84 (26.2)<br>AZA 67 (20.9)<br>Anti-TNF 28 (8.7)<br>MTX 14 (4.4)	During follow-up: 108 (33.6)	●Active IBD<br>●Longer IBD duration<br>●Prior surgery for IBD	Among those who developed NAFLD during follow-up:<br>7 (2.2)	NA
**Bosch, 2017 [34]**	Unicentric, Washington, USA	Cross-sectional study<br><br>Medical records review from university database	●IBD: 49<br>●IBD & PSC: 44<br>●IBD & PSC after LT:30	Histology<br>●Steatosis: 5-33% mild, 34-67% moderate, >67% severe<br>●NASH and fibrosis: NASH-CRN criteria	●IBD: 47 (43-51)<br>●IBD & PSC: 43 (38-47)<br>●IBD & PSC after LT: 47 (41-53)	●IBD: 25 (51)<br>●IBD & PSC: 31 (70)<br>●IBD & PSC after LT: 23 (75)	●IBD: 27.6 (25.1-30.2)<br>●IBD & PSC: 26.5 (24.8-28.1)<br>●IBD & PSC after LT: 25.7 (23.9-27.4)	●IBD: CD 33 (67)<br>UC 16 (33)<br>●IBD & PSC: CD 9 (20.5)<br>UC 35 (79)<br>●IBD & PSC after LT:	●IBD: SE 18 (37)<br>Anti-TNF 21 (43)<br>●IBD & PSC: SE 11 (24)<br>Anti-TNF 2 (5)<br>●IBD & PSC after LT: SE 14 (46)<br>Anti-TNF 2 (7)	NAFLD:<br>●IBD: 29 (59)<br>●IBD & PSC: 5 (11)<br>●IBD & PSC after LT: 1 (3)<br><br>NASH (% of the total patients of each group):<br>●IBD: 6 (12)	●Age<br>● BMI<br>●Diabetes<br>●Hypertension<br>●CD<br>●Absence of PSC	Cirrhosis:<br>●IBD: 4 (8)<br>●IBD & PSC: 19 (43)<br>●IBD & PSC after LT: 5 (17)	●No correlation between fibrosis stage and severity of steatosis

Table S1. Main characteristics of the 23 observational studies included in the systematic review

								CD 7 (23)<br>UC 23 (77)		●IBD & PSC: 0 (0)<br>●IBD & PSC after LT: 0 (0)			
**Carr, 2017 [35]**	Multicentric, USA	Retrospective cohort study<br><br>Medical records review	IBD and NAFLD: 84	●NAFLD: 3 patients (diagnostic codes), 80 (radiology), 1 (histology)<br>●FL: NAFLD fibrosis score	52.4±14.5	37 (44.1)	Obese 34 (47.9)	CD 60 (71.4)<br>UC 24 (28.6)	NA	-	IBD extent and severity do not predict NAFLD severity at time of NAFLD diagnosis	NAFLD fibrosis score - 2.03±0.244	●Metabolic syndrome<br>●Taking statins after NAFLD diagnosis (instead of prior to diagnosis)<br>●No influence of IBD treatments<br>●No influence of IBD extent and severity
**Carrillo-Palau, 2021 [36]**	Multicentric, Canary Islands, Spain	Cross-sectional study	●IBD: 151<br>●CG: 174 non-diabetic controls	●NAFLD: US<br>●LF: TE > 7.7 (>F2)	●IBD: 48±10<br>●CG: 50±16	●IBD: 65 (43)<br>●CG: 56 (32)	●IBD: 27±5<br>●CG: 30±3	CD 105 (70)<br>UC 46 (30)	5-ASA 53 (35)<br>Anti-TNF 48 (32)<br>AZA 47 (31)<br>IFX 28 (19)<br>ADA 20 (13)<br>MTX 20 (13)<br>Ustekinumab 6 (4)<br>Vedolizumab 5 (3)	●NAFLD in IBD:<br>Mild NAFLD: 40 (26)<br>Moderate NAFLD: 19 (13)<br>Severe NAFLD: 4 (2.6)	●Higher insulin, C-peptide and insulin resistance when NAFLD grade increased	●IBD: 5.0±2.0 kPa	●TE positively associated with insulin, C-peptide serum levels, insulin resistance and beta-cell function
**Chen, 2023**	Multicentric, UK	Prospective cohort study	Baseline:<br>●MAFLD:	●MAFLD: FLI ≥ 60	●MAFLD:	●MAFLD:	●MAFLD:	Incident IBD	NA	-	●MAFLD increased risk of	Incident IBD during follow-	●MAFLD with high risk of

Table S1. Main characteristics of the 23 observational studies included in the systematic review

**[37]**	(United Kingdom)	UK biobank Mean follow-up 12.1 years. Aim: detection of incident IBD	150,385 ●No MAFLD: 255,028	●Advanced LF: BAAT ≥2	57.4±7.8 ●No MAFLD: 56.1±8.2	95,363 (63.4) ●No MAFLD: 88,504 (34.7)	31.5±4.3 ●No MAFLD: 24.9±2.8	(cases/persons-years): ●MAFLD: 955/1,807,156 (CD 308, UC 647) ●No MAFLD :1273/3,109,082 (CD 377,UC 896)			incident CD, especially colonic and ileocolonic ●CD with perianal disease	up (cases/persons-years): ●Non-MAFLD: 1273/3,109,082 ●MAFLD not at high risk advanced LF: 105/224,621 ●MAFLD at high risk of advanced LF: 850/1,582,534	advanced LF, higher risk of CD, but not UC
**Kablawi, 2023 [38]**	Unicentric, Quebec, Canada	Cross-sectional study	IBD: 405	TE ●NAFLD: CAP≥275 dB/m ●NASH with LF: FAST score ●Significant LF: ≥ 8 kPa	45.1±15.3	186 (45.9)	25.8±5.0	CD 278 (68.6) UC 127 (31.4)	Anti-TNF 146 (36) 5-ASA 122 (30)	NAFLD 129 (31.9) NASH with LF: 11 (2.7% of IBD participants)	●NAFLD, longer IBD duration and UC were associated with intermediate-high CV risk	35 (8.6)	Significant LF was associated with intermediate-high CV risk
**Likhitsup, 2019**	Multicentric, Kansas,	Cross-sectional study	IBD: 80 (receiving anti-TNF)	●NAFLD: US ●NASH and	42±15	44 (55)	26±5.7	CD 65 (81) UC 15	Infliximab 67 (84) Adalimuma	●NAFLD: 43 (54) ●NASH	●Increased CDAI score ●Male sex	●LF: 7 (22) ●Cirrhosis: 0 (0)	NA

Table S1. Main characteristics of the 23 observational studies included in the systematic review

**[39]**	USA			LF: Fibrosure laboratory test. N0=0.25 no NASH, N1=0.50 probable NASH, N2=0.75 NASH				(19)	b 10 (13) Golimumab 2 (2.5) Certolizumab 1 (1.3) 5-ASA 12 (15) Thiopurines 17 (21) MTX 2 (2.5)	(N1+N2): 19 (23.8% of IBD participants, 61% of NAFLD participants with laboratory test)	●Higher BMI		
**Magri, 2019 [40]**	Unicentric, Monserrato, Italy	Cross-sectional study	IBD: 178	●NAFLD: US ●Significant LF: TE >7 kPa	●NAFLD: 53.9±12.9 ●No-NAFLD: 46.9±13.5	●NAFLD: 51 (52) ●No-NAFLD: 46 (48)	●NAFLD: 26.5±4.7 ●No-NAFLD: 23.9±13.7	CD: 83 (46.6) UC: 95 (53.4)	●NAFLD: SE 12 (27) Immunomodulators 11 (43) Biologic 28 (37) Surgery 11 (31) ●No-NAFLD: SE 31 (73) Immunomodulators 15 (53) Biologic 49 (63) Surgery 24 (69)	72 (40.4)	●Male sex ●Older age ●Obesity ●Metabolic syndrome ●Higher caloric and lipid intake	21 (16)	●Metabolic syndrome
**McHenry,**	Unicentric, St.	Cross-sectional,	●CD: 311 ●CG1: 622	NAFLD: liver	NA	NA	NA	CD: 311	NA	Absolute frequencies	●CD	Absolute frequencies	●CD

Table S1. Main characteristics of the 23 observational studies included in the systematic review

**2019 [41]**	Louis, USA	case control<br><br>CG1: internal controls, retrospective database of abdominal MR of St. Louis hospital<br><br>CG2: population-based sample from Dallas Heart Study	●CG2: 622	fat >5.5%<br><br>●CD and CG1: Magnetic Resonance-Proton Density Fat Fraction maps<br><br>●CG2: Magnetic Resonance Spectroscopy<br>LF: FIB-4 >1.3				(100)<br>UC: 0 (0)		not reported.<br>● CD: 38%<br>●CG1: 30% p=0.010<br>●CG2: 28% p=0.002<br><br>Lean (BMI<25 Kg/m^2):<br>● CD: 20%<br>●CG1: 10% p=0.024<br>●CG2: 10% p=0.013		not reported.<br>●CD: 11%<br>●CG2: 5%<br>p<0.001	
**Palumbo, 2018 [12]**	Unicentric, Montreal, Canada	Cross-sectional study	CD: 384	●NAFLD if CAP ≥248 dB/m, severe >292 dB/m<br>●Significant LF: ≥ 7 kPa (M probe) or ≥ 6.2 kPa (XL probe). Advanced fibrosis ≥8.7 and 7.2 kPa respectivel	●NAFLD: 50.2±12.8<br>●No-NAFLD: 38.7±14.9	●NAFLD: 65 (51.6)<br>●No-NAFLD: 108 (41.9)	●NAFLD: 28.2±4.9<br>●No-NAFLD: 23.5±3.7	CD 248 (64.6)<br>UC 136 (35.4)	5-ASA: NAFLD 43 (34.4), No-NAFLD 66 (25.6)<br>SE: NAFLD 9 (7.1), No-NAFLD 30 (11.6)<br>Thiopurines: NAFLD 23 (18.3), No-NAFLD 38 (14.7)<br>Anti-TNF:	NAFLD: 32.8%<br><br>Severe NAFLD: 19.3%	●Older age<br>●Higher BMI<br>●Higher triglyceride level	●Significant LF: 12.2% of CD patients, 24.6% of CD patients with NAFLD<br><br>●Advanced LF: 8.1 % of CD patients, 18.3% of CD patients with NAFLD	●Older age<br>●Older age at IBD diagnosis<br>●CD<br>●Higher BMI<br>●Hypertension<br>●Diabetes<br>●MTX (for advanced LF)

Table S1. Main characteristics of the 23 observational studies included in the systematic review

				y					NAFLD 45 (35.7), No-NAFLD 107 (41.5)<br>MTX: NAFLD 6 (4.8), No-NAFLD 3 (1.2)				
**Ribaldone, 2015 [43]**	Unicentric, Turin, Italy	Prospective cohort study<br><br>Mean follow-up 14 years. Aim: liver fibrosis development	CD: 35<br><br>Bowel surgery with contemporary liver biopsy at baseline	●Baseline: histology<br>●At the end of follow-up: TE, significant LF ≥ 7 kPa	35 (15-65)	21 (60)	NA	CD 35 (100)<br>UC 0 (0)	SE 33(94.3)<br>AZA 5 (14.3)	●Baseline, Steatosis: 9 (25)	NA	●Baseline: Fibrosis changes: 4 (11)<br>Significant LF: 0 (0)<br>●Follow-up: 5.2±1.2 kPa	●AZA<br>●Intermitent CD course
**Ritaccio, 2021 [42]**	Unicentric, Baltimore, USA	Retrospective cohort study<br><br>5 years follow-up (n= 56)	IBD and NAFLD 207 (selected from a cohort of 1,672 IBD patients)	Medical record review<br>●NAFLD: image exams and/or histology<br>●LF: NAFLD fibrosis score (-1.455-0.675 intermediate, >0.675 advanced	50.6±13.6	96 (46)	29.5±8.0	CD 131 (63)<br>UC 39 (19)<br>IC 9 (4)	Biologic 116 (56)<br>Immunosuppressants 106 (51)<br>5-ASA 334 (20)	Baseline:207 (12.4)	NA	Baseline (n= 136):<br>Indeterminate 42 (31)<br>Advanced LF or cirrhosis: 6 (4)<br><br>5 years (n= 56):<br>Indeterminate: 19 (34)<br>Advanced LF or cirrhosis: 5 (9)<br>9 (16) worsened, 4 (7) improved	●Average change in BMI in patients with LF worsening +1.3 Kg/m^2<br>●No effect of medication exposure

Table S1. Main characteristics of the 23 observational studies included in the systematic review

				LF or cirrhosis)								and 42 (77) stayed stable	
**Rodriguez-Duque, 2022 [16]**	Multicentric, Santander and Madrid, Spain	Cross-sectional, case control study	●IBD: 831<br>●CG: 1,718 (ETHON cohort)	TE<br>●Steatosis: CAP > 248 dB/m<br>●Significant LF: ≥7.2 kPa<br>●Advanced LF: ≥9.7 kPa	●IBD: 52 (19-76)<br>●CG: 51 (20-79)	●IBD: 401 (48.3)<br>●CG: 832 (48.4)	●IBD: 26.2±4.9<br>●CG:26.3±4.8	CD: 389 (46.8)<br>UC:420 (50.5)<br>IC: 22 (2.7)	Current SE: 25 (3)<br>Previous SE: 567 (68.2)<br>Current biologic:144 (17.3)<br>Previous biologic: 207 (24.9)<br>Current AZA: 147 (17.7)<br>Previous AZA: 348 (41.9)<br>Current MTX: 1 (0.1)<br>Previous MTX: 19 (2.3)<br>Previous surgery: 178 (21.4)	MAFLD<br>●IBD: 349 (42.0)<br>●CG: 563 (32.8)<br><br>p<0.001	MAFLD<br>●IBD<br>●IBD complications<br>●High BMI<br>●Older age<br>●Male sex<br>●Dyslipidemia	Advanced LF in patients with MAFLD:<br>(n=349)<br>●IBD: 33 (9.5)<br><br>(n=563)<br>●CG: 13 (2.3)<br><br>p<0.001	●IBD<br>●Crohn´s Disease<br>●IBD complications<br>●High BMI<br>●Type 2 Diabetes
**Sartini, 2018 [44]**	Unicentric, Modena, Italy	Retrospective, case-control study	●NAFLD + IBD: 78<br>●CG (NAFLD without IBD): 145	NAFLD: US review<br>LF: TE review (cut off not	●NAFLD + IBD: 51.2±11.8<br>●CG:54.	●NAFLD + IBD: 49 (62.8)<br>●CG: 43	●NAFLD + IBD: 28.6±4.8	CD 42 (53.8)<br>UC 36 (46.2)	5-ASA: 51 (65.4)<br>SE: 3 (3.8)<br>SE + 5-ASA: 4 (5.1)	-	NAFLD in IBD:<br>●Younger<br>●Lower BMI<br>●Less often hypertension, ↓	TE:<br>●NAFLD + IBD: 5.2±1.7<br>●CG: 6.4±3.5	NA

Table S1. Main characteristics of the 23 observational studies included in the systematic review

				specified) and APRI	9±12.5	(29.7)	●CG: 30.2±5.4		Thiopurines: 4 (5.1) Anti-TNF: 15 (19.2) Surgery: 27 (34.6)		HDL, ↑ fasting glucose and metabolic syndrome ●Severe IBD is associated with severe steatosis	APRI: ●NAFLD + IBD: 0.46±0.34 ●CG: 0.63±0.52	
**Simon, 2018 [11]**	Unicentric, Boston, USA	Cross-sectional study	CD: 462	NAFLD: CT LF: FIB-4 (advanced >1.45)	40±15	216 (46.7)	25.4±5.0	CD 462 (100) UC 0 (0)	NA	242 (52)	●Age ●Diabetes ●PNPLA3 genotype	Advanced LF: 32 (13.2% of patients with CD and NAFLD)	●IRGM rs13361189 CT/CC genotype ●IRGM rs4958847 AG/AA genotype
**Spagnuolo, 2019 [45]**	Unicentric, Catanzaro, Italy	Retrospective cohort study Mean follow-up 4 years. Aim: changes of steatosis severity and LF in patients who gained, lost or maintained weight	IBD: 89	Medical record review TE at baseline and during follow-up ●NAFLD: CAP >216 dB/m ●LF: >7 kPa	44±13	56 (63)	25±3	CD 29 (33) UC 60 (67)	5-ASA 83 (93) Anti-TNF 16 (18) Thiopurines 16 (18) SE 5 (6) MTX 1 (1)	Baseline: Liver steatosis: (66) Severe steatosis: (15) Mean difference of CAP after follow-up: ●↓ weight: -53±21 ●≈ weight: -2.1±18 ●↑ weight: 51±36	Weight gain is predictor of steatosis worsening	Baseline: LS 4.7±1 kPa Mean difference of LS after follow-up: ●↓ weight: -0.15±1 ●≈ weight: 0.21±1 ●↑ weight: 0.76±2	Significantly lower LS in patients who lost weight compared to those who gained weight
**Trifan,**	Unicentr	Cross-	IBD: 82	TE	49±13	45	25.3±4.	CD 37	5-ASA 43	38 (46.3)	●Older age	21 (25.7)	●Older age

Table S1. Main characteristics of the 23 observational studies included in the systematic review

**2022 [46]**	ic,Iasi, Romania	sectional study	●IBD + NAFLD: 38<br><br>●IBD without NAFLD: 44	●NAFLD: CAP ≥ 248 dB/m<br>●Significant LF: ≥7.2 kPa		(54.8)	7	(45.1)<br>UC 45 (54.9)	(52.5)<br>Biologic 24 (29.3)<br>AZA 9 (10.9)<br>SE 6 (7.3)		●BMI<br>●Longer IBD duration<br>●Age at diagnosis<br>●Bowel resection<br>●C-reactive protein<br>●Triglycerides<br>●Fasting plasma glucose	●IBD + NAFLD: 14 (36.9)<br><br>●IBD without NAFLD: 7 (15.9)	●Higher BMI<br>●IBD duration
**Van Lingen, 2021 [47]**	Unicentric, Leiden, The Netherlands	Prospective cohort study<br><br>Follow-up 6-12 months. Aim NAFLD and liver fibrosis prevalence	IBD: 82	TE<br>●Steatosis: CAP ≥ 248 dB/m<br>●Significant LF: ≥7.3 kPa	42.7±16.0	46 (56.1)	25.1±4.7	CD 40 (48.8)<br>UC 42 (51.2)	Previous surgery: 26 (32.1)	●Baseline: 32 (39.5)<br>●After follow-up: 29 (35.8)	●Increased waist circumference<br>●Increased CAP during follow-up if IBD activity	●Baseline: 16 (19.8)<br>●After follow-up:<br>10 (12.3)	●Increased LF during follow-up if IBD activity<br>●No other IBD related or metabolic risk factors
**Veltkamp, 2022 [48]**	Unicentric, Heidelberg, Germany	Cross-sectional study	IBD: 132	US and TE<br>●NAFLD: US and CAP ≥ 248 dB/m<br>●LF: ≥ 7 kPa	42 [31,57]	57 (43)	23 [20,26]	CD 79 (60)<br>UC 53 (40)	SE 112 (88)<br>Thiopurines 39 (31)<br>Anti-TNF 26 (20)<br>MTX 7 (6)	40 (30.3)	●Older age<br>●Higher BMI<br>●Longer IBD duration<br>●AST and GGT levels	11 (8)<br><br>7 of them had liver steatosis	●Longer IBD duration<br>●CD
**Yen, 2021 [49]**	Changhua, Taiwan	Retrospective cohort study	IBD: 81	US and TE<br>●NAFLD: CAP ≥ 248 dB/m<br>●Significant LF: >8kPa	43.5±14.4	58 (71.6)	22.4 [20.3, 25.6]	CD 36 (44.4)<br>UC 45 (55.6)	Anti-TNF 29 (35.8)<br>Anti-integrin 6 (7.4)<br>Previous surgery:19 (23.5)	24 (29.6)	●Higher BMI<br>●Older age at diagnosis<br>●Shorter IBD duration	4 (4.9)	NA

Table S1. Main characteristics of the 23 observational studies included in the systematic review

5-ASA: 5-Aminosalicylic acid. ADA: Adalimumab. APRI: AST to Platelet Ratio Index. AZA: Azathioprine. BAAT score: BMI, age, ALT, Triglycerides. CD: Crohn´s Disease. CDAI: Crohn´s Disease Activity Index. CT: Computed Tomography. CV: Cardiovascular. ETHON: Epidemiological sTudy of Hepatic infectiONs (Spanish Hepatitis C Prevalence Study). FAST score: Fibroscan-Aspartate Aminotransferase Score. FIB-4: Fibrosis 4 Index. HSI: Hepatic Steatosis Index. IBD: Inflammatory Bowel Disease. IC: Indeterminate Colitis. IFX: Infliximab. LF: Liver Fibrosis. LS: Liver Stiffness. MRI: Magnetic Resonance Imaging. MTX: Methotrexate. NA: Not Available. NAFLD: Non-Alcoholic Fatty Liver Disease. NASH-CRN: Non Alcoholic Steatohepatitis Clinical Research Network. TE: Transient Elastografy. UC: Ulcerative Colitis. LF: Liver Fibrosis. SE: Systemic Steroids. US: Abdominal Ultrasound. USA: United States of America. Continuous data are presented as mean ± Standard Deviation (SD), median (range) or median [Interquartile Range].
