# Supplementary material for: Liver Fibrosis in Non-Alcoholic Fatty Liver Disease and Progression to Hepatocellular Carcinoma in Patients with Inflammatory Bowel Disease: A Systematic Review
Source: Cancers (Basel). 2023 Jun 27;15(13):3367. doi: 10.3390/cancers15133367 (PMC10340150; doi:10.3390/cancers15133367)
Supplement: Supplementary file 1 [file cancers-15-03367-s001.zip › Tables S2-S4 Risk of bias assessment 5 april 2023.pdf]

Table S2. Risk of bias assessment of the 10 cross-sectional studies included in the systematic review according to the Newcastle-Ottawa Scale (NOS)

**Study, year**	**SELECTION**				**COMPARABILITY**	**OUTCOME**		**NOS total score (maximum 10 points)**
	**Representativeness of thesample**	**Sample size**	**Ascertainment of exposure**	**Non-respondents**		**Assessment of outcome**	**Statistical test**	
**Aggarwal, 2022 [32]**		✶	✶✶	✶	✶✶	✶✶	✶	**9**
**Bosch, 2017 [34]**			✶✶	✶	✶✶	✶✶	✶	**8**
**Carrillo-Palau, 2021 [36]**	✶	✶	✶✶	✶	✶✶	✶✶	✶	**10**
**Kablawi, 2023 [38]**	✶	✶	✶✶	✶	✶	✶✶	✶	**9**
**Likhitsup, 2019 [39]**	✶	✶	✶✶	✶	✶	✶✶	✶	**9**
**Magri, 2019 [40]**	✶	✶	✶✶	✶	✶	✶✶	✶	**9**
**Palumbo, 2018 [12]**	✶	✶	✶✶	✶	✶	✶✶	✶	**9**
**Simon, 2018 [11]**		✶	✶✶	✶	✶	✶✶	✶	**8**
**Trifan, 2022 [46]**	✶		✶✶	✶	✶✶	✶✶	✶	**9**
**Veltkamp, 2022 [48]**	✶	✶	✶✶		✶	✶✶	✶	**8**

NOS: Newcastle-Ottawa Scale. Single star: 1 point. Double stars: 2 points. A maximum of one star can be assigned to each item except for ascertainment of the exposure (selection), comparability and assessment of the outcome (outcome section), being the maximum total score 10 stars

Table S3. Risk of bias assessment of the 10 cohort studies included in the systematic review according to the Newcastle-Ottawa Scale (NOS)

**Study, year**	**SELECTION**				**COMPARABILITY**	**OUTCOME**			**NOS total score (maximum 9 points)**
	**Representativeness of the exposed cohort**	**Selection of the non-exposed cohort**	**Ascertainment of exposure**	**Outcome of interest not present at start of the study**		**Assessment of outcome**	**Length of follow-up period**	**Adequacy of follow-up**	
**Abomhya, 2022 [10]**		✶	✶	✶	✶	✶	✶	✶	**7**
**Al-Mohannadi, 2020 [31]**	✶		✶	✶		✶	✶	✶	**6**
**Bessisow, 2016 [33]**	✶		✶	✶		✶	✶	✶	**6**
**Carr, 2017 [35]**	✶		✶	✶		✶	✶		**5**
**Chen, 2023 [37]**	✶	✶	✶	✶	✶✶	✶	✶	✶	**9**
**Ribaldone, 2015 [43]**			✶	✶		✶	✶	✶	**5**
**Ritaccio, 2021 [42]**	✶	✶	✶		✶	✶	✶		**6**
**Spagnuolo, 2019 [45]**	✶	✶	✶	✶	✶✶	✶	✶	✶	**9**
**Van Lingen, 2021 [47]**	✶		✶	✶		✶	✶	✶	**6**
**Yen, 2021 [49]**	✶		✶	✶		✶			**4**

NOS: Newcastle-Ottawa Scale. Single star: 1 point. Double stars: 2 points. A maximum of one star can be assigned to each item except for comparability, which can be assigned two stars (maximum total score 9 stars).

Table S4. Risk of bias assessment of the 3 case-control studies included in the systematic review according to the Newcastle-Ottawa Scale (NOS)

**Study, year**	**SELECTION**				**COMPARABILITY**	**EXPOSURE**			**NOS total score (maximum 9 points)**
	**Case definition adequate?**	**Representativeness of the cases**	**Selection of controls**	**Definition of controls**		**Ascertainment of exposure**	**Same method for cases and controls**	**Non-response rate**	
**McHenry, 2019 [41]**	✸		✸	✸	✸✸	✸			**6**
**Rodriguez-Duque, 2022 [16]**	✸	✸	✸	✸	✸	✸	✸	✸	**8**
**Sartini, 2018 [44]**		✸	✸		✸	✸	✸	✸	**6**

NOS: Newcastle-Ottawa Scale. Single star: 1 point. Double stars: 2 points. A maximum of one star can be assigned to each item except for comparability, which can be assigned two stars (maximum total score 9 stars).
